# Synthesis of Samarium-Cobalt Sub-micron Fibers and Their Excellent Hard Magnetic Properties

**DOI:** 10.3389/fchem.2018.00018

**Published:** 2018-02-07

**Authors:** Jimin Lee, Tae-Yeon Hwang, Min Kyu Kang, Hong-Baek Cho, Jongryoul Kim, Nosang V. Myung, Yong-Ho Choa

**Affiliations:** ^1^Department of Materials Science and Chemical Engineering, Hanyang University, Ansan, South Korea; ^2^Department of Chemical and Environmental Engineering, University of California, Riverside, Riverside, CA, United States

**Keywords:** samarium cobalt, electrospinning, fiber, permanent magnet, magnetic properties

## Abstract

High-throughput synthesis of Samarium-Cobalt sub-micron fibers with controlled composition and dimension was demonstrated by combining electrospinning and reduction-diffusion processes. The composition of fibers was readily varied (8 < Sm < 20 at.%) by adjusting precursor composition whereas the diameter of fibers was precisely controlled by varying electrospinning parameters (e.g., applied voltage, solution feed rate, temperature, and humidity) to reach single-domain size. X-ray diffraction patterns confirmed that single phase Sm_2_Co_17_ fibers were synthesized when the metal precursor ratio (Sm^3+^/(Sm^3+^+Co^2+^)) was precisely controlled at 10.6%, whereas mixed phases (i.e., Co-Sm_2_Co_17_ or Sm_2_Co_17_-Sm_2_Co_7_) were observed when the ratio is deviated from the stoichiometric. Magnetic saturation (*M*_*s*_) of the synthesized fibers monotonically decreased with an increased in Sm content. In contrast, coercivity (*H*_*c*__i_) monotonically increased with an increase in Sm content.

## Introduction

Rare-Earth/Transition-Metal (RE-TM) permanent magnets such as Nd-Fe-B, Sm-Co, and Sm-Fe-N are essential part in a wide range of applications including direct current (DC) rotating electric motors in automobiles, data storage, magnetoelectronic, electromechanical, and electronic devices (Campbell, 1996; Liu et al., 2008). Among these RE-TM permanent magnets, Sm-Co based alloy magnets are the promising materials for high-temperature applications due to excellent magnetocrystalline anisotropy constant (approaching 17.0 × 10^6^ J/m^3^) and the higher Curie temperatures (*T*_*c*_ of ~1,190 K) (Strnat, 1990; Pan, 2014).

Recently, as the devices are becoming miniaturized and high efficiency, more enhanced magnetic performance of magnetic materials is necessarily required. Some researchers predicted that enhanced hard magnetic properties (e.g., coercivity) can be achieved when the dimension of materials reaches the single-domain size (e.g., theoretical single domain size for Sm_2_Co_17_ = 0.66 micron and SmCo_5_ = 1.6 micron) (Jiles, 2003; Hadjipanayis and Prinz, 2013; Hou and Sellmyer, 2017). At this condition, the magnetic spin in each single-domain of particle gives highest resistance to demagnetization, leading to greater coercivity. The other way to enhance hard magnetic properties is to create high aspect ratio structures which will increase shape anisotropy (Park et al., 2000; Lu et al., 2007; Zabel and Farle, 2012; Han et al., 2014). That is to say, the enhanced hard magnetic properties are predicted from one-dimensional Sm-Co sub-micron fibers.

Sm-Co nano- and micro- structures have been fabricated by various methods including ball-milling (Liu and McCormick, 1999; Zheng et al., 2012; Wang et al., 2013), co-precipitation (Zhang et al., 2011), sol-gel process (Suresh et al., 2012), and polyol process (Saravanan et al., 2011a). However, these methods are difficult to control the dimension including diameter and length due to heterogeneous nucleation and growth. Electrospinning is a scalable nanomanufacturing process where the dimension (e.g., diameter from tens of nm to several microns) and morphology can be readily controlled by adjusting precursor solution composition and electrospinning parameters (Huang et al., 2003; Barakat et al., 2008; Shuakat and Lin, 2014).

**Figure 1:**
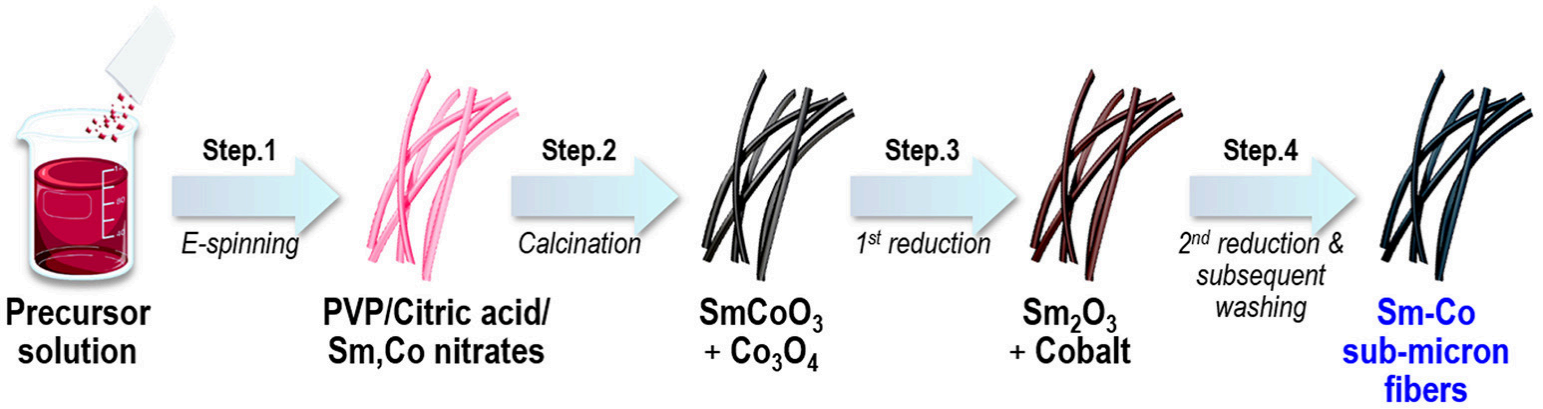
Schematic drawing of the process flow.

In this work, we demonstrated to ability to synthesize Sm-Co fibers with controlled composition and dimension by combining electrospinning and reduction-diffusion process. Figure 1 shows a schematic representation of fabrication processes where electrospinning and several annealing process were subsequently carried out to synthesize fibers.

## Materials and methods

### Materials

The raw materials for these experiments were samarium(III) nitrate hexahydrate [Sm(NO_3_)_3_·6H_2_O, 99.9%; Sigma-Aldrich, USA], cobalt(II) nitrate hydrous [Co(NO_3_)_2_·6H_2_O, 99.9% up; Kojundo Chemical, Japan], Polyvinylpyrrolidone (PVP, M_w_≈1,300,000; Sigma-Aldrich, USA), citric acid anhydrous (99.5% up; DAEJUNG Chemical & Metals Co., Ltd., South Korea), and calcium hydride (CaH_2_, 92%; Alfa Aesar, England). All chemicals were used without further purification.

### Preparation of Sm-Co fibers

The ratio of Sm(NO_3_)_3_·6H_2_O to Co(NO_3_)_2_·6H_2_O were varied from 8, 10.6, 13, 16.7, and 20 at.% of Sm. These salts were dissolved in a mixed solvent of 3 mL of deionized water and 1 mL of ethanol followed by acoustic mixing under 60 Hz for 10 min. Blends were prepared in a laboratory scale mixer (PharmaRAM™ I Mixer, Resodyn Corporation, USA) for homogeneous mixing. An appropriate amount of PVP were added into the solution to reach the PVP concentration of 4.0 wt.%. The viscosity and the electrical conductivity of all the prepared solutions were kept within 118~120 cP and 26.5~27.5 mS/cm, respectively. Ten milliliters of precursor solution was loaded into a plastic syringe with a 30-gauge needle. The needle was connected to a high voltage power supply, faced vertically to the rotating drum collector. The specific process conditions were: the applied voltage of 20 kV, the distance between the needle tip and the collector was15 cm, the solution feeding rate was 0.3 mL/h. Temperature and relative humidity were 30°C and below 20% of relative humidity, respectively. The spun fibers were dried overnight at 80°C to remove solvent residue. The dried fibers were then calcined at 900°C for 2 h in a box furnace under ambient air to decompose organics including polymer. The calcined fibers were 1st reduced at 700°C for 2 h in pure H_2._ Finally, the as-reduced fibers were mixed with CaH_2_ as a reducing agent, and subsequently 2nd reduced at 700°C for 2 h under argon environment. The customized stainless steel (SUS304) crucible with the close-fitting cover was utilized as the reaction chamber to minimize the loss of volatile Sm source and to keep the sufficient retention time for the reaction. (See also description over the SUS304 crucible in Results and discussion) To remove residual CaH_2_ and byproducts after the reduction, the products were washed several times with the 0.1 M of dilute acetic acid solution, deionized water and ethanol, then dried in an oven at 60°C.

### Characterization

Thermal gravimetric analysis (TGA, SDT Q600, TA Instruments) was employed to study thermal behavior of the as-spun and metal oxide fibers. The analysis was carried out with a heating rate of 10°C/min up to 1,000°C, in Air and H_2_, respectively. The phase and crystallographic characteristics of the fibers were identified using an X-ray diffractometer (XRD, D/MAX-2500/PC, Rigaku) with Cu Kα radiation (1.5406 Å). Field-emission scanning electron microscopy (FE-SEM, MIRA-3, Tescan) and transmission electron microscopy (TEM, JEM-2100F, JEOL) were employed to analyze the morphology and microstructure. The quantitative elemental contents were measured by transmission electron microscopy energy-dispersive X-ray spectroscopy (TEM-EDS, JEM-2100F, JEOL) and X-ray Fluorescence Spectrometer (XRF, ZSX Primus II, Rigaku). Magnetic properties were measured at room temperature using a physical property measurement system (PPMS, PPMS-9T, Quantum Design) with a maximum applied field of 90 kOe.

## Results and discussion

### Optimization of calcination process condition

**Figure 2:**
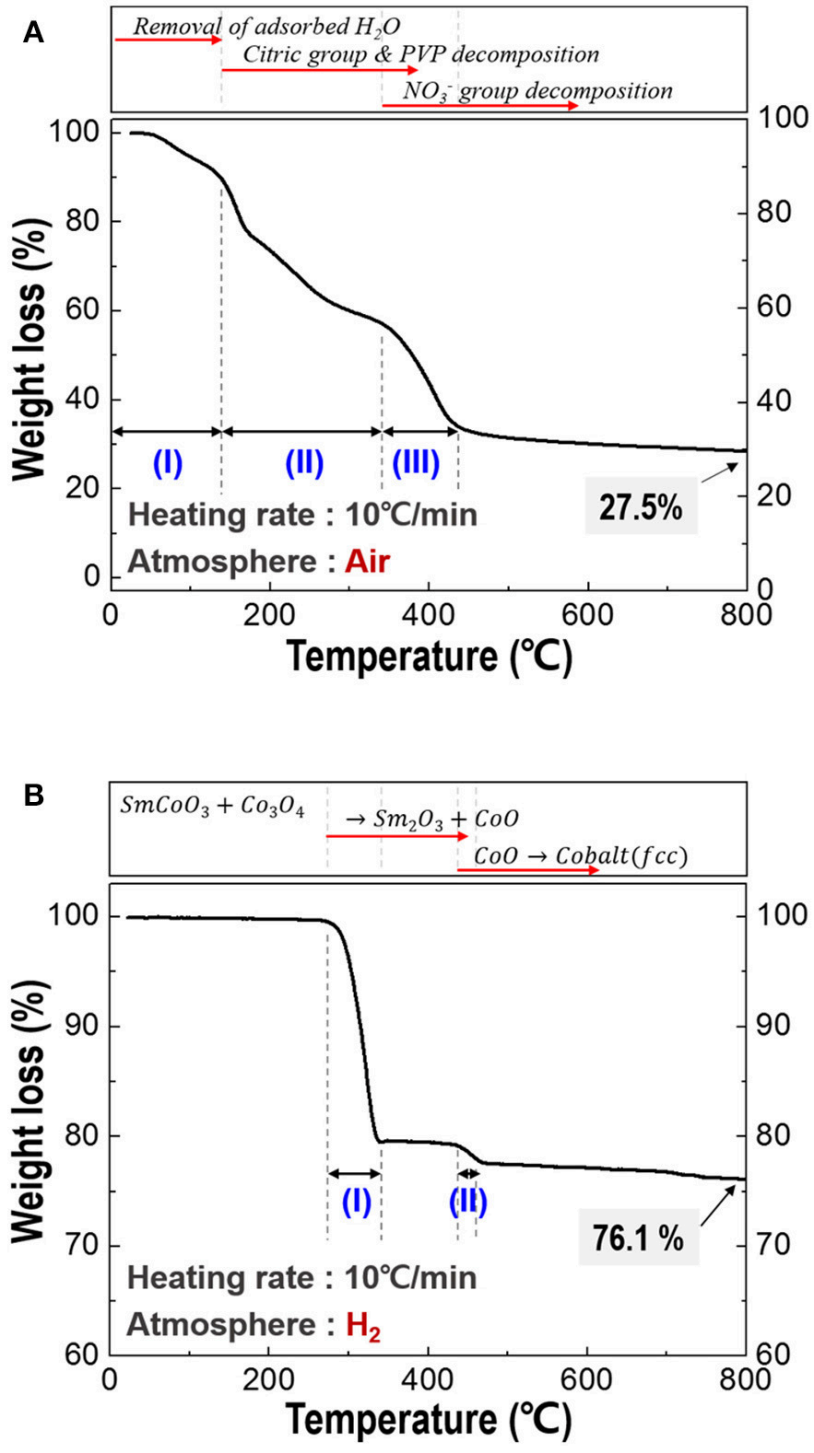
Thermogravimetric analysis (TGA) of the composite fiber; **(A)** spun fibers composed of PVP/Sm nitrate/Co nitrate/citric acid, and **(B)** calcined fiber which is made up of SmCoO_3_/Co_3_O_4_.

For the optimization of heating temperature, the TGA thermograms of the spun PVP/nitrates/citric acid composites and calcined fibers was investigated and shown in Figure 2. As shown in Figure 2A, there are many stages in TG curve, named stage (I), stage (II), and stage (III). At stage (I), the mass loss was attributed to removal of the adsorbed water. At stage (II), most of the organic materials including citric group and PVP were degraded. ${\text{NO}}_{3}^{-}$ group finally decomposed in stage (III). Through decomposition behavior of the spun fiber, all the organics were expelled and SmCoO_3_-Co_3_O_4_ phases were synthesized (Keely and Maynor, 1963; Barbooti and Al-Sammerrai, 1986; Loría-Bastarrachea et al., 2010; Melnikov et al., 2014). Total weight loss was about 72.5% at below 800°C. Thus, the calcination temperature was selected at 800°C to make sure all the organics decompose. The TGA curve for the calcined nanofibers (Figure 2B) showed two clear degradation stages; the first stage was the phase transition from the initial SmCoO_3_-Co_3_O_4_ phases through an intermediate mixture of Sm_2_O_3_-CoO when heated to 330°C (Kelly et al., 2016). The second step was the conversion of CoO to metallic cobalt when heated above 430°C (Olusola and Sudip, 2016). Temperature above 500°C is selected as an appropriate reduction temperature and the total weight loss was about 22.9% up to 500°C.

According to previous works, rare-earth element oxides such as Sm_2_O_3_, Gd_2_O_3_, and Nd_2_O_3_ are difficult to be reduced to metallic form unless the operating temperature is greater than 1,000°C, which is consistent with our observation where no degradation in TG curve of the as-reduced fiber sample was observed under H_2_ atmosphere till 1,000°C (data not shown) (Gupta and Krishnamurthy, 2013). It was reported that rare-earth metals were obtained from their oxide form at low temperature through reduction-diffusion process by employing calcium or CaH_2_ as a reducing agent which is based on the difference of the free energy between oxide materials (Cech, 1974; Sharma, 1986; Machlin, 2010). Table 1 lists the Gibbs free energy (Δ*G*^0^) of CaO and Sm_2_O_3_ at 298 and 1,000 K, respectively. (Yoon, 2013) The reduction proceeds as described in Equation (1):

(1) $$\begin{array}{r} \frac{1}{3}Sm_{2}O_{3}+Ca\to \frac{2}{3}Sm+CaO \end{array}$$

(2) $$\begin{array}{r} \Delta G^{0}=G_{CaO}^{0}-G_{{\frac{1}{3}Sm}_{2}O_{3}}^{0} \end{array}$$

**Table 1:** Free energy of CaO and Sm_2_O_3_.

**Oxides**	***ΔG*_298__K_ (J)**	***ΔG*_1, 000__K_ (J)**
1/3 Sm_2_O_3_	−5.73 × 10^5^	−5.11 × 10^5^
CaO	−6.04 × 10^5^	−5.33 × 10^5^

From Equation (2), because Δ*G*^0^ < 0 in the temperature range 298 to 1,000 K, the reaction (Equation 1) occurred spontaneously and thus Sm_2_O_3_ could be fully reduced even at low temperature. (Burrows et al., 2017) Drawing on this, CaH_2_, which decomposes into Ca and H_2_ under inert condition, was selected as a reductant in our work and 700°C was selected enabling the low temperature reduction.

### Phase, morphology, and magnetic properties of Sm-Co nanofibers

**Figure 3:**
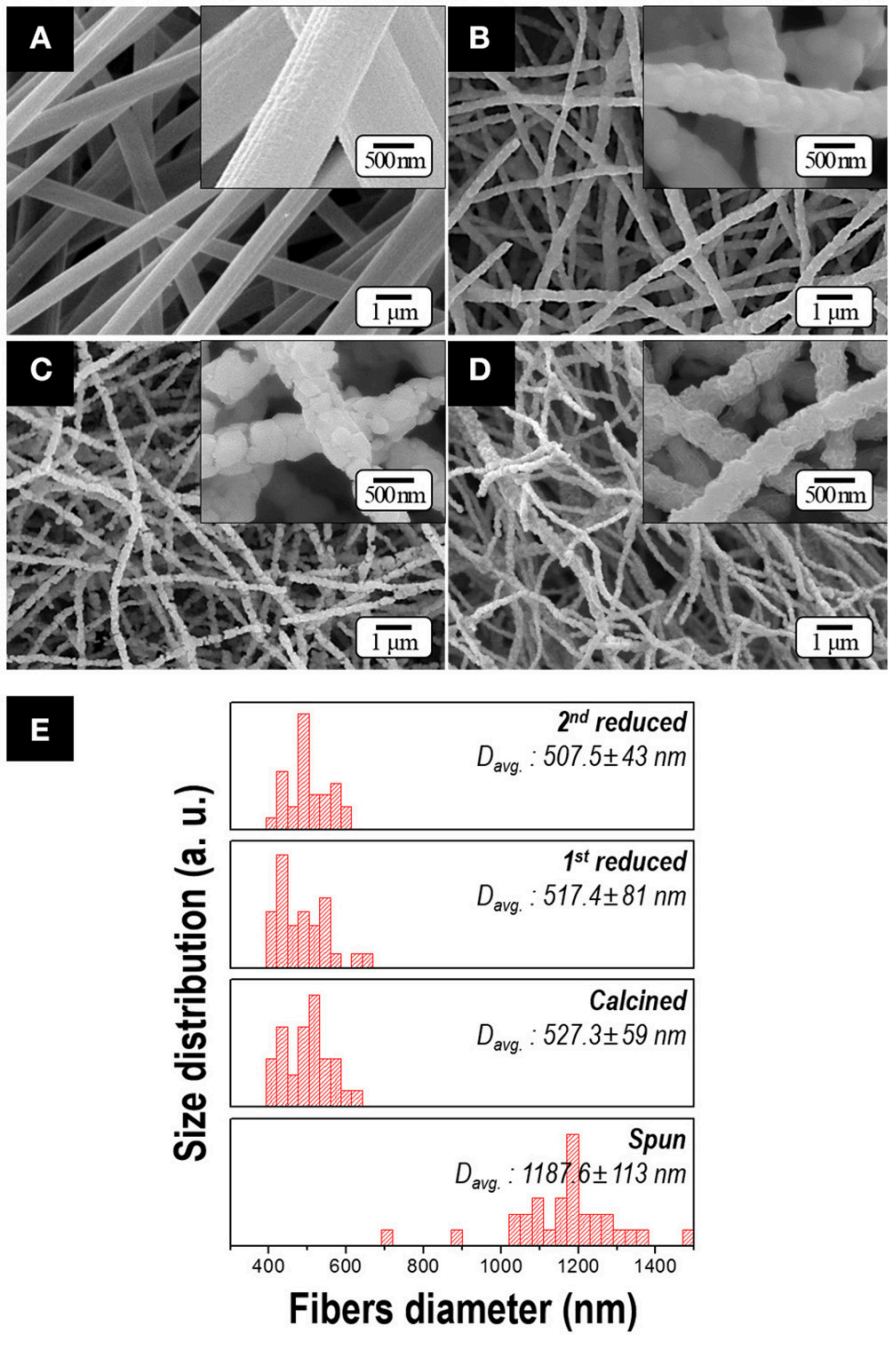
FE-SEM micrographs of samples obtained after; **(A)** electrospinning, **(B)** calcination, **(C)** 1st reduction and **(D)** 2nd reduction with subsequent washing (Insets: high magnification images). The size distribution of fiber diameters for various samples obtained at each process is presented in **(E)**. All the samples are prepared by utilizing the precursor solution containing 10.6 at.% of Sm.

Figure 3 shows the morphologies of the samples obtained after each process. Citric acid was employed to obtain soft and uniform morphology of the spun fibers resulting from the formation of cobalt(II) citrate complex [i.e., $3{\text{Co}}^{2+}+{2C}_{6}H_{5}O_{7}^{3-}\to {\text{Co}}_{3}{\left(C_{6}H_{5}O_{7}\right)}_{2}$], which has the higher moisture resistance than cobalt nitrate in the atmosphere (Lee et al., 2016). Owing to this additive, the uniform and bead-free spun fibers were obtained with an average diameter of 1 μm as shown in Figure 3A. After calcination, as all of the organic materials were expelled, the diameter of nanofiber decreased to nearly half (Figure 3B). After 1st and 2nd reduction process, the 500-nm-diameter fibers with an aspect ratio of >50 were observed in Figures 3C–E. There is no notable difference in dimension or morphology regardless of the Sm composition in the precursor solution. It is because the range of diameters of the fibers strongly depends on the viscosity and electrical conductivity of the precursor solutions and these values were kept within the specific range in our study (Cramariuc et al., 2013).

**Figure 4:**
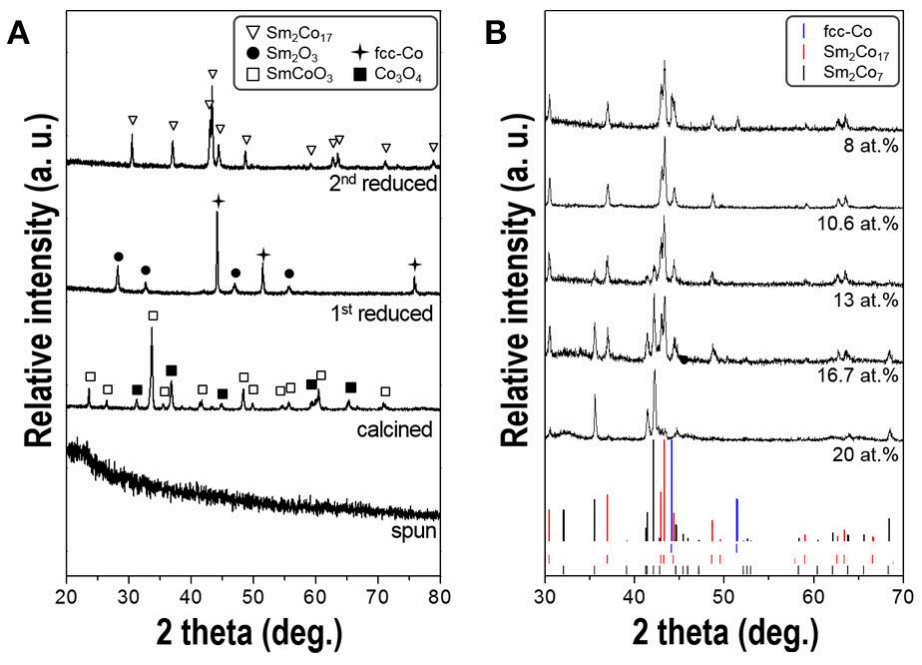
**(A)** XRD patterns of the synthesized fibers obtained after each process; electrospinning, calcination, 1st reduction, and 2nd reduction, respectively. All the composition of samples is 10.6 at.% of Sm. **(B)** XRD patterns of 2nd reduced nanofibers having different Sm atomic contents ranging from 8 to 20 at.%.

Figure 4A shows the XRD patterns of the fibers obtained after each process. The as-spun fibers mainly comprised of nitrates, citric acid and PVP show the typical amorphous structure. After calcination at 800°C, SmCoO_3_ (JCPDS No.70-4511) and Co_3_O_4_ (JCPDS No.43-1003) phases were generated with high crystallinity. The SmCoO_3_-Co_3_O_4_ nanofibers were then reduced to Sm_2_O_3_ (JCPDS No.15-0813) and face-centered-cubic cobalt [(fcc)-Co] (JCPDS No.77-7452) phases when subjected to 1st reduction under pure H_2_ condition. After final 2nd reduction at 700°C and subsequent washing steps, only hexagonal Sm-Co phases were obtained without any byproduct patterns such as CaO. Thermodynamically, Sm only reacts with Co then generates Sm-Co intermetallic compound due to the low solubility of Sm and Co in Ca as well (Deng et al., 2010).

The XRD patterns for the synthesized Sm-Co fibers with different atomic percent of Sm, ranging from 8 to 20 at.%, are shown in Figure 4B. It exhibits the coexistence of the mixtures of Sm_2_Co_7_ (JCPDS No.58-0293; 22.2 at.% of theoretical Sm) and Sm_2_Co_17_ (JCPDS No.65-7762; 10.6 at.% of Sm), without SmCo_5_ (;16.7 at.% of Sm) phase. According to the Sm-Co equilibrium phase diagram, (Khan, 1974) Sm_2_Co_17_ and Sm_2_Co_7_ phases are more stable at the reduction temperature of 700°C than SmCo_5_ within the range from 0 to 25 at.% of Sm. Because the eutectoid temperature of SmCo_5_ is about 805°C, (Perry, 1976) the sample annealed at 800°C shows an existence of SmCo_5_ phase but the morphology was not maintained to be one-dimensional and degraded into agglomerated particles which was not in accordance with the direction we pursued. (data not shown) The more intense Sm_2_Co_17_ diffraction peaks were observed when the Sm content decreased while Sm_2_Co_7_ peaks almost disappeared. In the sample with 10.6 at.% of Sm, only Sm_2_Co_17_ pattern was observed implying there is no Sm loss in whole processes. Some researchers reported that a small amount of Sm loss occurs during the heating and/or washing process then they used up to 40% of excess Sm source (Lin et al., 1996; Hou et al., 2007; Lee et al., 2011; Zhang et al., 2011; Yoon, 2013). It can be considered that using the customized crucible with close-fitting cover prevents losing volatile Sm (Spedding, 1960). (Supplementary Figure 1) A few peaks of metallic cobalt (JCPDS No.77-7452) were detected in the sample with 8 at.% of Sm. The presence of residual cobalt is attributed to the insufficient amount of Sm source to form a Co-rich phase of Sm_2_Co_17_.

**Figure 5:**
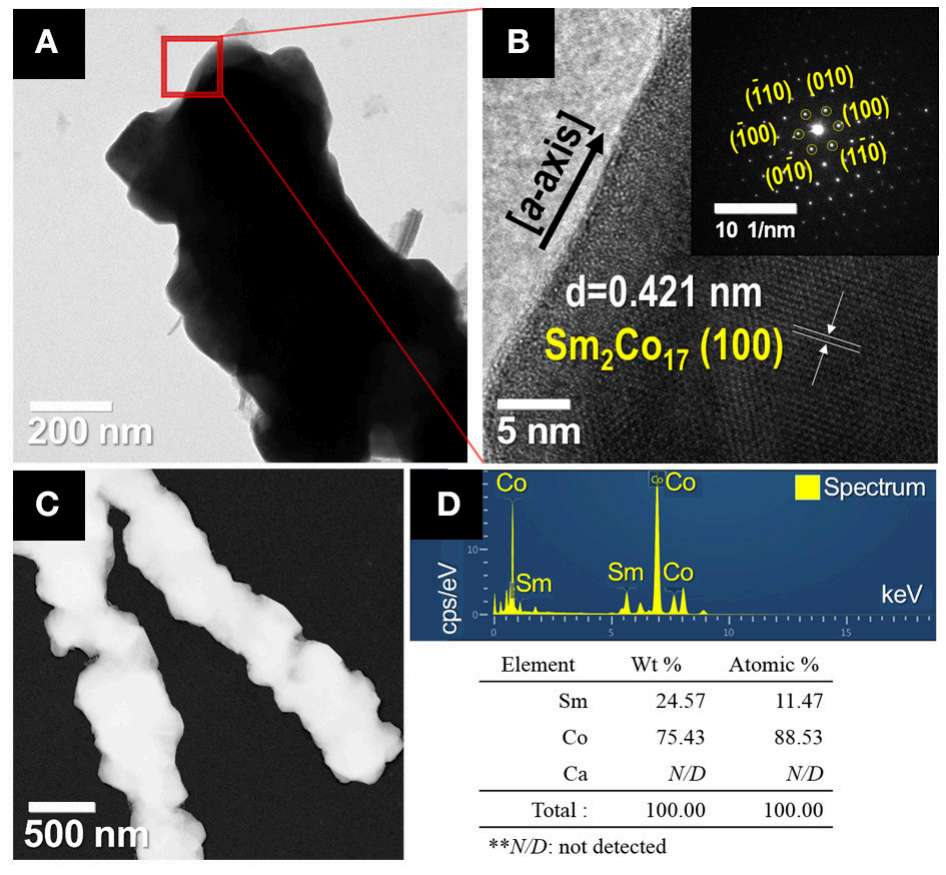
TEM images of synthesized Sm_2_Co_17_ nanofibers; **(A)** low magnification and **(B)** HR-TEM micrograph and SAED pattern as indicated by the red square in **(A)**. **(C)** Bright-field STEM image of the synthesized Sm_2_Co_17_ fibers and **(D)** TEM-EDS spectrum of Sm_2_Co_17_ fibers with chemical compositions of the constituents as determined by TEM-EDS.

The microstructures of the synthesized Sm_2_Co_17_ fibers were analyzed by TEM, as shown in Figure 5. The high-resolution TEM (HR-TEM) image of a part of the Sm_2_Co_17_ fibers shows the inter-planar spacing of about 0.421 nm, corresponding the (100) lattice projections of the hexagonal structure of Sm_2_Co_17_ crystal (JCPDS No.65-7762). Selected area electron diffraction (SAED) pattern reveals the sharp diffraction spots, implying the presence of single- and high-crystalline Sm_2_Co_17_ with growth direction in *a*-axial (the inset image of Figure 5B).

The impurity profiles including Ca were investigated using TEM-EDS and XRF analysis (Figures 5C,D and Supplementary Table 1) The EDS data shows the presence of samarium and cobalt without any impurities. XRF analysis confirmed the presence of Sm and Co without trace elements (i.e., < 0.1 wt.%).

**Figure 6:**
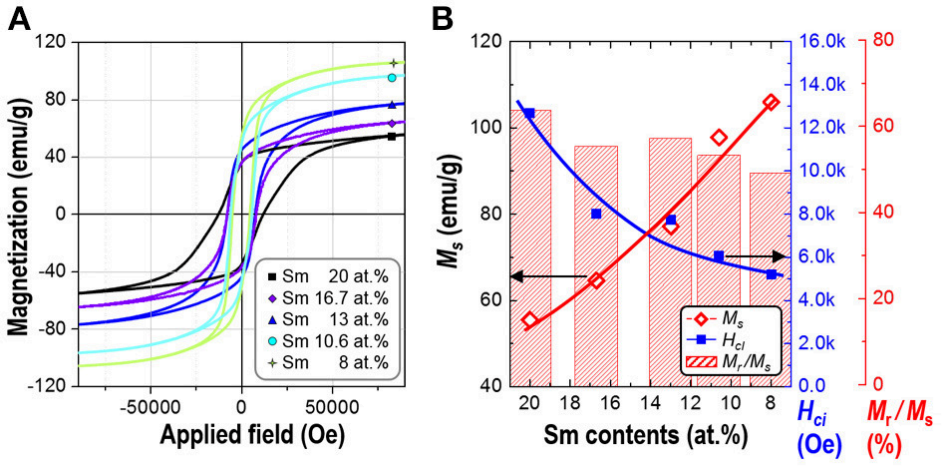
**(A)** Magnetic hysteresis loops and **(B)** the change of saturation magnetization (*M*_*s*_), squareness (*M*_*r*_/*M*_*s*_), and intrinsic coercivity (*H*_*ci*_) of synthesized Sm-Co fiber with different Sm contents (at.%).

Magnetic properties of the synthesized powders of Sm-Co sub-micron fibers were characterized using PPMS without compaction and sintering process. Figure 6 shows the magnetic hysteresis loops of the samples as a function of the Sm content. The corresponding values of saturation magnetization (*M*_*s*_), remanence (*M*_*r*_), squareness (*M*_*r*_/*M*_*s*_), and coercivity (*H*_*ci*_) are given in Table 2. All the samples demonstrated hard magnetic behaviors irrespective of their phase composition. As the Sm content decreases, *H*_*ci*_ also decreases from 12,676 to 5,210 Oe. On the contrary, *M*_*s*_ monotonically increases which is attributed to the increase of the volume fraction of Sm_2_Co_17_ (Kumar, 1988). It is well-known that Sm_2_Co_17_ phase has lower magnetocrystalline anisotropy (*K*_a_ = 3.5 × 10^6^ J/cm^3^) and higher saturation magnetization (*M*_*s*_ = 100 emu/g); whereas Sm_2_Co_7_ phase exhibits the higher *K*_a_ and the lower *M*_*s*_, 6.3 × 10^6^ J/cm^3^ and 0.79 T, respectively (De Campos et al., 1998; Saravanan et al., 2011b). The increase in the *M*_*s*_ from 10.6 to 8 at.% of Sm is mainly attributed to the existence of metallic cobalt showing a soft ferromagnetism. The *M*_*s*_ value of bulk cobalt is reported about 162.55 emu/g (Nishikawa et al., 1993). For the single phase of Sm_2_Co_17_ fibers, *M*_*s*_ was within 98% of theoretical value for Sm_2_Co_17_ alloy at room temperature (Werner, 1969). It indicates that Sm_2_Co_17_ grains in the fibers were well-crystallized and there is no or few impurities exist leading to the low magnetic properties.

**Table 2:** The values of saturation magnetization (*M*_*s*_), remanence (*M*_*r*_), squareness (*M*_*r*_/*M*_*s*_), and coercivity (*H*_*ci*_) for the synthesized samples.

**Sm content (at.%)**	***M_*s*_*(emu/g)**	***M_*r*_*(emu/g)**	***M_*r*_/M_*s*_*(%)**	***H_*ci*_*(Oe)**
8.0	106.0	52.4	49.5	5,210
10.6	97.8	52.5	53.7	6,066
13.0	77.2	44.4	57.6	7,750
16.7	64.6	36.0	55.7	8,020
20.0	55.5	35.6	64.1	12,676

Table 3 compares these results with other reported data (Saravanan et al., 2011a,b; Zhang et al., 2011; Zheng et al., 2012; Yang et al., 2014; Cui et al., 2015). The higher *M*_*s*_ can also be explained due to the well-crystallized Sm_2_Co_17_ grain and low impurity contents less than 0.1 wt.% including Ca as a byproduct, and minimum oxide phase on the surface of Sm-Co. The magnetic properties of the electrospun sub-micron fibers were also greater than nanowires prepared by using electrochemical fabrication. The large *H*_*ci*_ is attributed to the appropriate dimension of fiber, near single-domain size of Sm_2_Co_17_ (Hadjipanayis and Prinz, 2013). When the size decreases from single-domain size, the magnetic properties drastically drops and finally shows the superparamagnetic behavior (Tian et al., 2012; Hou and Sellmyer, 2017).

**Table 3:** Magnetic properties of some earlier reported Sm_2_Co_17_ nanostructures.

**Structure**	**Size (nm)**	**Synthesis method**	**Magnetic properties**			**References**
			***M_*s*_*(emu/g)**	***M_*r*_*/*M_*s*_***	***H_*ci*_*(kOe)**	
Nanoparticle (0-D)	*D_*avg*._*: 6	Co-precipitation	~50.0	0.80	5.8	Zhang et al., 2011
	*D_*avg*._*: 90	Co-precipitation	74	<0.10	0.24	Saravanan et al., 2011b
	*D_*avg*._*: 100	Polyol process	85.3	<0.20	1.05	Saravanan et al., 2011a
	*D_*avg*._*: 18	Ball-milling	–	>0.75	4.7	Zheng et al., 2012
Nanowire (1-D)	*D_*avg*._*: 50 *L:* > 2,000	Electrochemical fabrication	–	0.39	0.817	Yang et al., 2014
	*D_*avg*._*: 50 *L:* 12,000	Electrochemical fabrication	–	>0.9	<2.5	Cui et al., 2015
	*D_*avg*._*: 500 *L:* >25,000	Electrospinning	97.8	0.54	6.1	This work

## Conclusion

In summary, Sm-Co sub-micron fibers with average diameter of 500 nm were successfully fabricated *via* combined process of electrospinning and reduction-diffusion process. These combined processes produce high quality Sm-Co fibers with controlled morphology and composition. Synthesized fibers showed the excellent hard magnetic properties which were attributed to the high shape anisotropy from high aspect ratio morphology and near single-domain size structure by controlled diameter of fiber. These processes provide cost-effective routes to fabricate high quality high aspect ratio hard magnetic fibers with controlled morphology and composition.

## Author contributions

JL prepared the manuscript and figures. T-YH and MK helped the analysis and interpretation of the data. H-BC and JK helped and advised on this manuscript. NM and Y-HC contributed equally to this manuscript and accepted responsibility for conduct of research and final approval.

### Conflict of interest statement

The authors declare that the research was conducted in the absence of any commercial or financial relationships that could be construed as a potential conflict of interest. The reviewers FZ, ZZ, and handling Editor declared their shared affiliation.
