# Supplementary material for: Synthesis of Samarium-Cobalt Sub-micron Fibers and Their Excellent Hard Magnetic Properties
Source: Front Chem. 2018 Feb 7;6:18. doi: 10.3389/fchem.2018.00018 (PMC5808290; doi:10.3389/fchem.2018.00018)
Supplement: Supplementary file 1 [file Table1.pdf]

Supplementary Material

Synthesis of Samarium-Cobalt sub-micron fibers and Their Excellent Hard Magnetic Properties

Jimin Lee, Tae-Yeon Hwang, Min Kyu Kang, Hong-Baek Cho, Jongryoul Kim, Nosang V. Myung* and Yong-Ho Choa*

*** Correspondence:** Yong-Ho Choa: choa15@hanyang.ac.kr
Nosang V. Myung: myung@engr.ucr.edu

Supplementary Table 1. Chemical composition of the synthesized Sm_2Co_{17} nanofibers as determined by XRF.

	Co	Sm	Fe	P	Ca	S	Si	Cr
**Mass %**	75.156	24.441	0.093	0.081	0.075	0.068	0.051	0.035
