# Supplementary material for: Synthesis of Samarium-Cobalt Sub-micron Fibers and Their Excellent Hard Magnetic Properties
Source: Front Chem. 2018 Feb 7;6:18. doi: 10.3389/fchem.2018.00018 (PMC5808290; doi:10.3389/fchem.2018.00018)
Supplement: Supplementary file 2 [file Image1.pdf]

Supplementary Material

Synthesis of Samarium-Cobalt sub-micron fibers and Their Excellent Hard Magnetic Properties

Jimin Lee, Tae-Yeon Hwang, Min Kyu Kang, Hong-Baek Cho, Jongryoul Kim, Nosang V. Myung* and Yong-Ho Choa*

*** Correspondence:** Yong-Ho Choa: choa15@hanyang.ac.kr
Nosang V. Myung: myung@engr.ucr.edu

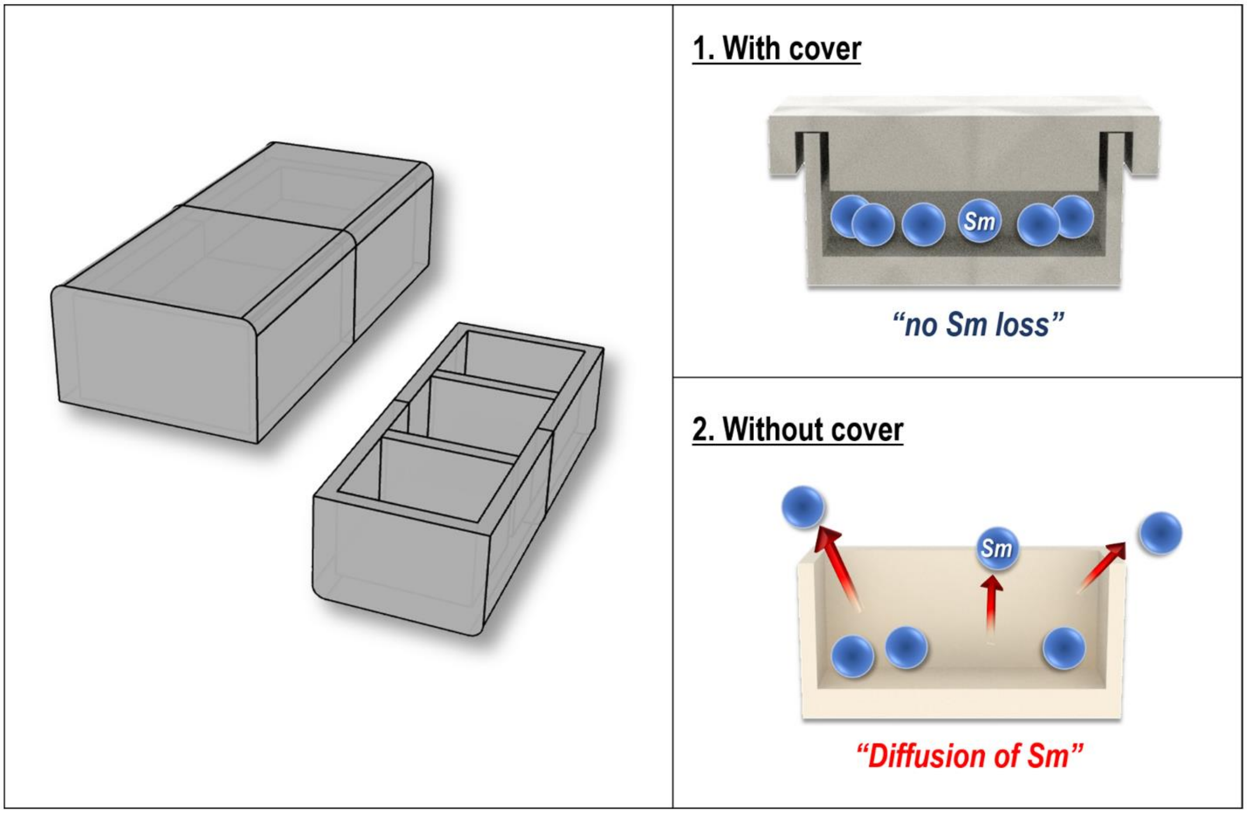

Supplementary Figure 1. (A) Drawings of the customized stainless steel (SUS304) crucible with close-fitting cover. The size of the crucible used is 30 mm(W) × 95 mm(L) × 30 mm(H).
